# A PGPR-Produced Bacteriocin for Sustainable Agriculture: A Review of Thuricin 17 Characteristics and Applications

**DOI:** 10.3389/fpls.2020.00916

**Published:** 2020-07-07

**Authors:** Mahtab Nazari, Donald L. Smith

**Affiliations:** Department of Plant Sciences, McGill University, Montreal, QC, Canada

**Keywords:** plant growth promoting rhizobacteria bacteriocins, *Bacillus thuringiensis* NEB17, anti-microbial activity, signal molecules, phytomicrobiome

## Abstract

A wide range of prokaryotes produce and excrete bacteriocins (proteins with antimicrobial activity) to reduce competition from closely related strains. Application of bacteriocins is of great importance in food industries, while little research has been focused on the agricultural potential of bacteriocins. A number of bacteriocin producing bacteria are members of the phytomicrobiome, and some strains are plant growth promoting rhizobacteria (PGPR). Thuricin 17 is a single small peptide with a molecular weight of 3.162 kDa, a subclass IId bacteriocin produced by *Bacillus thuringiensis* NEB17, isolated from soybean nodules. It is either cidal or static to a wide range of prokaryotes. In this way, it removes key competition from the niche space of the producer organism. *B. thuringiensis* NEB17 was isolated from soybean root nodules, and thus is a member of the phytomicrobiome. Interestingly, thuricin 17 is not active against a wide range of rhizobial strains involved in symbiotic nitrogen fixation with legumes or against other PGPR. In addition, it stimulates plant growth, particularly in the presence of abiotic stresses. The stresses it assists with include key ones associated with climate change (drought, high temperature, and soil salinity). Hence, in the presence of stress, it increases the size of the overall niche space, within plant roots, for *B. thuringiensis* NEB17. Through its anti-microbial activity, it could also enhance plant growth *via* control of specific plant pathogens. None of the isolated bacteriocins have been examined as broadly as thuricin 17 on plant growth promotion. Thus, this review focuses on the effect of thuricin 17 as a microbe to plant signal that assists crop plants in managing stress and making agricultural systems more climate change resilient.

## Introduction

Microbes produce antimicrobial substances to compete with each other for nutritional resources and niche space. These excreted microbial substances comprise a range of types: broad-spectrum non-ribosomal antibiotics, metabolic products (organic acids), lytic agents (lysozymes), and bacteriocins (Riley, 1998). Bacteriocins are ribosomally synthesized antibacterial peptides secreted by bacteria (Arnison et al., 2013). They are distinct for antibiotics in that they inhibit organisms closely related to the producer strains, active at very low concentrations and are formed in the ribosome (Mak, 2018). Bacteriocins are bactericidal and/or bacteriostatic – inhibiting growth of target organisms – depending on identity, growth conditions, growth stage of the target strain, and on bacteriocin concentration (Nes et al., 2006). Most bacteria, Gram-negative or Gram-positive produce at least one type of bacteriocin; archaea may produce bacteriocin-like antimicrobials known as archaeocins (Riley and Wertz, 2002; Cotter et al., 2005). The “bacteriocin” concept was introduced in 1953. The proteinaceous nature of bacteriocins means they can be degraded in the digestion system of animals, allowing them to be used as natural preservatives in foods (Cleveland et al., 2001). Most research around bacteriocins has been conducted on lactic acid bacteria (LAB), known for their biopreservative potential in the food industry, and often produced by “generally recognized as safe” (GRAS) microbial strains (O’sullivan et al., 2002). While bacteriocins can be effective biocontrol agents in the food industry and medicines, less effort has been focused on their potential for agricultural application. Simultaneously, there is the need to reduce the negative effects of chemical fertilizers, herbicides, and pesticides on the environment, with a view to achieving environmentally sustainable agriculture. The main objective of this review is to summarize the characteristics of thuricin 17 and provide knowledge regarding its efficacy in plant growth promotion and resistance to abiotic stresses. We attempt to illuminate the promising possibility of bacteriocins as biostimulant agents for the agriculture sector.

## Biosynthesis and Mode of Action of Bacteriocins

**Figure 1:**
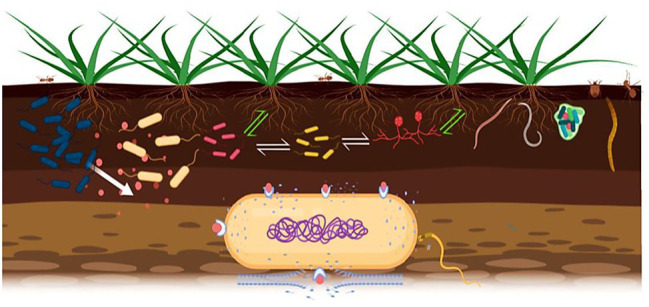
Signal exchanges in the phytomicrobiome are indicated; white arrows for microbe-to-microbe signals and green arrows for plant-to-microbe and microbe-to-plant signals. Bacteriocins can act as microbe-to-microbe and microbe-to-plant signal molecules as well. Producer strain (blue color) excretes bacteriocin against closely related strains. The bacteriocins bind to the transport/receptor proteins in the outer membrane of the target cell and pass through it by various mechanisms, then form pores that result in efflux of proteins, ATP, and ions, causing cell death.

Most bacteriocins are synthesized as biologically inactive peptides with an N-terminal leader peptide, holding the molecule in an inactive configuration. The N-terminal sequence plays a major role in interactions with the excretory apparatus and is also recognized by enzymes responsible for modifications, in the case of post-translationally modified bacteriocins (Cotter et al., 2005). However, a growing number of newly identified bacteriocins lack leader sequences and are active immediately after translation (Fujita et al., 2007). Specific immune proteins, encoded in the genome, are required for expression of bacteriocins, allowing the producer bacterial cell to resist the bacteriocin action (Rameshkumar et al., 2016). Production of bacteriocins and immune proteins is often mediated by quorum-sensing mechanisms, which may also be induced by environmental stressors (Nes and Eijsink, 1999). The killing mechanism for most bacteriocins is pore formation in cell membranes and enzyme activity, particularly nucleases against DNA, rRNA, and tRNA (Bizani et al., 2005; Chavan and Riley, 2007; Gillor and Ghazaryan, 2007). Bacteriocins are strongly cationic peptides that easily bind to the membrane bilayer of negatively charged phospholipids. The interaction between bacterial target membranes and the hydrophobic elements of bacteriocins produces non-specific ionic channels; pore formation causes leakage of intracellular components, such as ions, ATP, and small proteins, collectively leading to cell death (Bharti et al., 2015; Figure 1). Modes of bacteriocin action are related to the peptide’s primary structure. Since classification of bacteriocins is based on structure, bacteriocins belonging to the same class have similar modes of action (Iwatani et al., 2011).

## Bacteriocin Classification

Bacteriocins excreted by Gram-negative bacteria typically fall into four categories based on their size; to be specific: colicins, colicin-like bacteriocins, microcins, and phage tail-like bacteriocins (Chavan and Riley, 2007). Bacteriocins of Gram-positive bacteria are more discussed in more detail as our target bacteriocin, thuricin 17 is produced by a Gram-positive bacterium. These bacteriocins are grouped into four classes, based on their genetic and biochemical characteristics or the presence of post-translational modifications, molecular weight, heat stability, proteolytic enzyme stability, presence of disulfide or monosulfide bonds, and cidal method: (1) Class I are small post-transitionally modified bacteriocins (<5 kDa, 19–37 amino acids) containing the unusual amino acids lanthionine and methyllanthionine (hence the name lantibiotics) that have negative or no net charge and target indispensable catalytic enzymes of vulnerable species (Deegan et al., 2006), and they are heat stable peptides and target the skeleton of the cell wall (Sahl and Bierbaum, 1998; González-Martínez et al., 2003). This class is divided to subclasses *Ia* and *Ib*: the first being positively charged bacteriocins that kill by pore formation. Nisin, a member of this class, is the only bacteriocin regarded as safe for human consumption (McAuliffe et al., 2001; Gharsallaoui et al., 2016). The second subclass includes bacteriocins with rigid, globular structures acting by inhibition of catalytic enzymes required for peptidoglycan synthesis in target cells (Ditu et al., 2014). (2) Class II bacteriocins are heat-stable with molecular weights less than 10 kDa, and they are non-modified and distinguished by a hydrophilic N-terminal sequence (Heng et al., 2007). This class can be subclassified into four groups: subclass *IIa*, is the largest subclass, which includes antilisterial bacteriocins such as pediocin PA-1, with large potential in food preservation as well as medical use (Rodríguez et al., 2002; Fimland et al., 2005). Subclass *IIb* consists of multi-component bacteriocins requiring at least two different peptides, further subdivided into synergistic (S) and enhancing (E) inhibitory agents (Marciset et al., 1997; Flynn et al., 2002; Garneau et al., 2002). Subclass *IIc* are circular bacteriocins requiring cysteine residues for activity (Joerger and Klaenhammer, 1986). Subclass *IId* is comprised of one-peptide, linear bacteriocins possessing specific cidal methods related to their diversity of fundamental structures (Iwatani et al., 2011; Cotter et al., 2013). The bacteriocin focused on in this review, thuricin 17, is categorized in the latter subclass: a small single peptide with a molecular weight of 3.162 kDa and sharing N-terminal homology (DWTXWSXL) with bacteriocin F4 synthesized by *Bacillus thuringiensis* spp. kurstaki strain BUPM4 (Kamoun et al., 2005). The similar amino acid sequence of these two bacteriocins indicates a possible specific role of bacteriocins of this sequence and similar modes of action (Gray et al., 2006b). (3) Class III includes large peptides (>30 kDa), which are divided into heat-labile lytic bacteriocins, lysing the bacteria cell wall in an enzymatic manner, and heat-labile non-lytic bacteriocins that disturb glucose transfer or metabolism (Joerger and Klaenhammer, 1986). (4) Class IV are complex circular bacteriocins with lipid or carbohydrate moieties making them susceptible to glycolytic or lipolytic enzymes (Lewus and Montville, 1991; Wirawan et al., 2007).

## Rhizosphere Microbiome Bacteriocins

Of the various microbial populations present in the rhizosphere, bacteria are the most abundant microorganisms (Kaymak, 2010). Bacteriocin producer strains can be present in the rhizosphere, and some strains are plant growth promoting bacteria (PGPR; Subramanian and Smith, 2015). A diverse group of signal molecules (microbe-to-plant, plant-to-microbe, and microbe-to-microbe) are exchanged in the rhizosphere (Figure 1) and govern the establishment of successful plant-microbe relationships (Smith et al., 2015). Plant-associated bacteria use bacteriocins as non-self-propagating suppressive agents causing hostility between closely related strains (Tagg et al., 1976), and bacteriocinogenic activity has been detected in nearly all rhizobial species (Triplett and Sadowsky, 1992) and plays a significant role in the phytomicrobiome. A narrow body of studies has taken shape around ecological impacts of bacteriocins in natural environments, with native strains. For example, production of multiple R-tailocins by *Pseudomonas chlororaphis* 30-84 is considered to be a competitive approach that contributes to the persistence of the producer strain in the wheat rhizosphere microbiome, as compared to bulk soil, perhaps there could be more bacterial interaction in the rhizoplane due to the greater population and nutrient availability (Dorosky et al., 2018). However, most research only focus on pairwise interactions of a bacteriocin producer and a target strain in a culture assay. The capacity for bacteriocin excretion by PGPR is reported, such as *Pseudomonas fluorescens* SF39a, isolated from the wheat rhizosphere secreting bacteriocins that inhibits the growth of the phytopathogenic *Pseudomonas* and *Xanthomonas* strains (Godino et al., 2016). “Rhizobiocins” are bacteriocins synthesized by rhizobia (Schwinghamer, 1975) such as production of bacteriocin-like substances from *Bradyrhizobium japonicum* and other slow-growing rhizobia (Gross and Vidaver, 1978), some rhizobial strains associated with *Medicago* and *Rhizobium leguminosarum* bv. *viciae* (Wilson et al., 1998; Hafeez et al., 2005). It has also been reported that *R. leguminosarum* strains possess symbiotic plasmid pRL1J, which contains essential nodulation and nitrogen fixation genes as well as determinants for secretion of small, medium, or large bacteriocins (Schwinghamer and Brockwell, 1978; Hirsch, 1979; Hirsch et al., 1980). Interestingly, some bacteriocins play a significant role in nodulation competitiveness against specific strains. For example, molecular features and biological characteristics of rhizobiocin, produced by *R. leguminosarum* 248, provide nodulation competitive advantage over specific strains, either recently isolated or wild types ones (Oresnik et al., 1999). *Bacillus* strains produce antimicrobial substances, including peptides and lipopeptides, antimicrobials, and bacteriocins. The small bacteriocin cerin 7, produced by *Bacillus cereus*, was the first reported bacteriocin-like compound from a *Bacillus* species (Oscáriz et al., 1999). Many bacteriocins occur in the rhizosphere, for instance cerein8A from *B. cereus* (Bizani et al., 2005), Bac-GM17 from *Bacillus clausii* GM17 (Mouloud et al., 2013), H4, IH7, and Bac14B from *Bacillus subtilis* (Compaoré et al., 2013), that have potential for agricultural application. Because of a wide range of proteins it excretes, *B. thuringiensis* is the most studied among *Bacillus* species; it can be easily separated from closely related species by its ability to produce natural insecticides against diptera, coleoptera, and lepidoptera larvae (Schnepf et al., 1998; Palma et al., 2014). Until now, synthesis of 18 bacteriocins from *B. thuringiensis* have been reported (Mojgani, 2017), such as thuricinS, thuricin7, entomocin110, morricin269, and tochicin (Cherif et al., 2001, 2008; Chehimi et al., 2007; De la Fuente-Salcido et al., 2008). However, bacteriocin production by *Bacillus* PGPR is poorly understood and none have been studied for plant growth promotion as extensively as thuricin 17, discovered in our laboratory (Smith et al., 2008) and produced by *B. thuringiensis* NEB17 (BtNEB17), a non-symbiotic endophytic bacterium isolated from soybean root nodules. Co-inoculation of this strain with *B. japonicum* 532C promoted soybean root nodulation, plant growth, and yield (Bai et al., 2003). Subsequently, a compound was isolated from the growth medium in which BtNEB17 was cultivated, and named thuricin 17 (Gray et al., 2006a).

## Characteristics of Thuricin 17: A Novel Bacteriocin From Class *Iid*

Thuricin 17 is synthesized during mid-exponential growth and continues through to the stationary phase, thus it would seem to be a secondary metabolite. The nucleotide sequence of the gene region encoding thuricin 17 indicated that there are three copies of the gene synthesizing this bacteriocin. There have been, over time, changes in the nucleotide sequences of the three genes, but all the changes are at the third codon position and code for redundancies, so that the genes all code for the same protein, suggesting constraints on evolution of the genes (Lee et al., 2009). The dual function (bacteriocin and microbe-to-plant growth promoting molecular signal) nature of this protein might be the constraint: it both inhibits a range of bacteria and triggers plant growth (Lee et al., 2009). To understand the antimicrobial activity of thuricin 17, a range of *Bacillus* and *non-Bacillus* species were studied; results indicated no inhibitory effect on nodulating rhizobia and other PGPR strains (Gray, 2005). However, thuricin 17 acts as inhibitor to *Escherichia coli*, a unique finding regarding this peptide since it is uncommon for Gram-positive bacteria to inhibit Gram-negative bacteria (Gray et al., 2006a). Thuricin 17 is highly resistant to denaturation between −20 and 100°C, and is biologically stable across a pH range of 1.0–9.25 (Gray et al., 2006a). Bacteriocins can have a cysteine residue in the C terminus, among the last three positions, which can permit formation of a disulfide bridge, allowing folding of the peptide into a cluster (Oscáriz and Pisabarro, 2001). The presence of four cysteine residues in thuricin 17 allows for the possible formation of two disulfide bridges. This might be the reason for stability of this peptide to extreme temperatures and pH levels (Gray et al., 2006b). The mode of action of the bacterial peptide is both bactericidal and bacteriostatic (Gray, 2005). *B. cereus* ATCC 14579 has been observed to manifest a static effect whereby *B. thuringiensis* spp. *thuringiensis* Bt 1627 was able to recover and showed delayed growth, suggesting that the mechanism involved either degradation of a lethal peptide or that there had been a shift in gene expression to allow resumption of growth (Gray et al., 2006a).

## Potential Role of Thuricin 17 As a Plant Biostimulant

Bacteriocin excretion provides producer strains with an advantage, through significant reduction of direct competitor populations, allowing improved performance and survival of the producer strain. PGPR producing bacteriocins benefit from this competitive ability to inhibit closely related strains and thus clearing the niche space for themselves (Riley and Wertz, 2002). A bacteriocin that also promotes plant growth and development through mechanisms such as a decrease in the population of root associated plant-bacterial pathogens, would result in more vigorous plants (Subramanian and Smith, 2015). However, another advantage that extracellular PGPR (ePGPR) could provide is exemplified by the bacteriocin producing *B. thuringiensis* NEB17 which was shown to have no harmful effects on nodulating rhizobia and a range of other known PGPR, such as *Serratia proteomaculans* 1-102, 2-68, *Pseudomonas putida*, and other *Bacillus* species such as, *Bacillus licheniformis* Alfa-Rhiz and *B. subtilis* NEB 5 and NEB4 (Gray, 2005). Perhaps, because various species of PGPR occupy different niches in the rhizosphere, less interspecies competition occurs among them, such as rhizobial PGPR occupying the interior of nodule cells (Rachwał et al., 2016), versus *Bacillus* PGPR present in the nodule cortex (Spratt, 1919). Thus, bacteriocins may target PGPR types, which are likely to compete with the producer strain most directly, and often these are closely related strains with similar physiologies and requirements. In this way, the bacteriocin expands available niche space for the producer strain by eliminating potential competitors.

Positive correlations, indicating potential positive interactions, between ePGPR bacteriocin production and nodulation by intracellular PGPR (iPGPR) indicate another mechanism of plant growth promotion (Prudent et al., 2015). Co-inoculation of *B. thuringiensis* NEB17 with *B. japonicum*, isolated from soybean root nodules, enhanced soybean nodulation (Bai et al., 2003). Thuricin 17, produced by *B. thuringiensis* NEB17, increases plant growth through direct and indirect mechanisms (Table 1). Indirect mechanisms of action for this “signal” molecule include induction of plant disease resistance (Mabood et al., 2014) and inhibition of susceptible pathogenic strains by binding to receptors or the cell membrane/wall, leading to an increase in ecological niche space for producer strains or nodulation of associated plants (Gray and Smith, 2005).

**Table 1:** Examples of thuricin 17 application to promote plant growth and resistance to abiotic stresses.

Crop	Growth condition	Thuricin 17 mode of action	Reference
*Arabidopsis thaliana*	Salt stress	More than two fold changes in activation of some important carbon, energy, and antioxidant metabolism pathway proteins including PEP-carboxylase, Rubisco-oxygenase, and pyruvate kinase, leading to mitigation of stressful conditions	(Subramanian et al., 2016b)
*A. thaliana*	Salt stress	Increased levels of IAA (85%) and SA (42%) and decreased gibberellins, cytokinins and jasmonate, causing amelioration to salt stress	(Subramanian, 2014)
Corn (*Zea mays*)	Non-stressful	Enhanced leaf area and dry weight at 3 leaf stage	(Lee et al., 2009)
Canola [*Brassica napus* (L.)]	Stressful cold temperature and salinity	Promoted dry biomass and root development	(Schwinghamer et al., 2016a)
Soybean (*Glycine max*)	Non-stressful	Induced defense system: enhanced production of liginification-related enzymes and their isoforms, peroxidase and superoxide dismutase enzymes (antioxidative enzymes)	(Jung et al., 2008, 2011)
Soybean	Non-stressful	Provided competitive advantage to the nodulating stain when thuricin 17 was applied as root-drench on inoculated plants with *Bradyrhizobium japonicum* 532C so nodule number, root and shoot total biomass increased; foliar application also enhanced leaf area, leaf greenness, and shoot N concentration	(Lee et al., 2009)
Soybean	Water stress	Enhanced abscisic acid (ABA) levels in leaves and roots leading to root elongation which increased water and nutrient uptake	(Prudent et al., 2015)

Treatment with thuricin 17 enhanced production of phenolics, phenylalanine ammonia lyase activity (lignification-related enzymes), and also the levels of peroxidase and superoxide dismutase enzymes (antioxidative enzymes) in 2-week old soybean plants, indicates that it provoked defense system responses (Jung et al., 2008, 2011). Direct stimulation takes place when this compound binds to receptors in leaf or root tissues, and acts as a pseudo-stress signal leading to triggering of various metabolic pathways, resulting in enhancement of photosynthetic rates. Although thuricin 17 is quite stressful to some prokaryotes, it may induce a pseudo-stress response in plants (Gray, 2005). Generally, plants elevate photosynthetic rates under pathogen or insect challenge, to compensate for decreased photosynthesis in damaged tissues (Nowak and Caldwell, 1984). In the case of thuricin 17, the response has been induced without any stress to counteract, resulting in a net increase in growth (Gray and Smith, 2005). When thuricin 17 was root-drench-applied nodule number root, shoot, and total biomass of soybean was increased; foliar application also enhanced leaf area, leaf greenness, and shoot N concentration (Lee et al., 2009). Similarly, leaf area and dry weight of corn and soybean seedlings were enhanced by thuricin 17 treatment, indicating that this signal molecule is effective on both C3 dicot and C4 monocot species (Lee et al., 2009).

Research on thuricin 17 has demonstrated its promising role as a plant growth promoter under stressful conditions. As an example, thuricin 17 treated soybean plants showed a reduced impact by water deficit stress; application of thuricin 17 to soybean roots plus inoculation with N_2_-fixing *B. japonicum* increased root and nodule biomass by 37 and 55%, respectively and also increased leaf area, photosynthetic rate, and abscisic acid levels in roots under water deficit stress (Prudent et al., 2015). Canola [*Brassica napus* (L.)] showed a positive response to thuricin 17 treatment, which caused reconfiguration of leaf arrangement plus enhanced biomass production and root development in peat pellets and plant culture vessel growth systems, under stressful temperatures and salinity conditions (Schwinghamer et al., 2016a). Only canola seeds treated with thuricin 17 developed roots under very stressfully low temperature (10/4°C) and salt stress conditions (Schwinghamer et al., 2016a). Likewise, plants treated with thuricin 17 produced one more leaf per plant than the control treatment and other biostimulant treatments at 30/30°C, which is very stressful for a temperate zone crop such as canola (Schwinghamer et al., 2016b). Rosettes of *Arabidopsis thaliana* Col-0 treated with thuricin 17 had decreased levels of cytokinins, gibberellins, jasmonic acid, and abscisic acid at 24 h after treatment with thuricin 17, and increased levels of indole-3-acetic acid (IAA; 85%) and salicylic acid (SA; 42%) compared to controls (Subramanian, 2014).

A proteomic study indicated more rapid and efficient mobilization of carbon, nitrogen, and storage proteins of soybean seeds treated with thuricin 17, resulting in enhanced germination under salt stress (Subramanian et al., 2016a). Treatment of 3-week old *A. thaliana* plants with thuricin 17 resulted in alteration of carbon and energy metabolism pathways under unstressed and salt stress conditions: PEP carboxylase, rubisco-oxygenase, pyruvate kinase, and proteins of the light harvesting complex, energy and antioxidant pathways were all increased by thuricin 17 treatment, mitigating salt stress effects (Subramanian et al., 2016b). Collectively, these findings highlight the role of thuricin 17 as a microbe-to-plant signal stimulating plant growth, particularly under conditions of environmental stress. Thuricin 17 is the only bacteriocin examined in such depth. Currently, we are conducting studies to discover full mode of action of thuricin 17, and its role in mitigation of either abiotic or biotic stress; for the latter as biocontrol agents, we are still unsure as only *in vitro* antagonism experiments have been conducted and results are still unpublished. To be a successful biocontrol agent, the bacteriocin needs to be examined in plant, to compete with phytopathogens. If the results are promising, thuricin 17 would be of great interest for commercial application.

## Conclusions

Global demand for agricultural produce is on the rise and the productivity of crops must be increased, even in the face of developing climate change conditions. Biomolecules produced by PGPR are of great interest in this capacity. Overall, thuricin 17 acts as a signal molecule to promote plant growth and development, particularly under stressful conditions, through a range of mechanisms; changes in carbon, energy, and antioxidant metabolism pathway protein activities, induction of synthesis of enzymes related to plant defense systems, increases in photosynthetic rate, stimulated production of some phytohormones such as IAA, SA, and ABA, and modification of the root system to better uptake of water and nutrients. The potential role of PGPR excreted compounds that are both bacteriocins and plant growth promoters presents inspiring possibilities and research opportunities. They stimulate plant growth, in part through alleviation of abiotic stress effects and could allow more sustainable management in agriculture, plus increased resilience to climate change conditions. More studies should be conducted to elaborate the biocontrol potential/impact of the compound, examining the ecological role of thuricin 17 in the natural environment and working to discover the thuricin 17 receptor, to determine its action on signaling pathways within plant cells.

## Author Contributions

MN assembled the literature and developed a first version of the manuscript. DS provided the initial perspective and provided editorial and conceptual input as the manuscript development progressed. All authors contributed to the article and approved the submitted version.

## Conflict of Interest

The authors declare that the research was conducted in the absence of any commercial or financial relationships that could be construed as a potential conflict of interest.
